# A 10–20 GHz 6-Bit High-Accuracy Digital Step Attenuator with Low Insertion Loss in 0.15 µm GaAs p-HEMT Technology

**DOI:** 10.3390/mi15010084

**Published:** 2023-12-30

**Authors:** Ding He, Zhentao Yu, Jie Chen, Kaiyuan Du, Zhiqiang Zhu, Pu Cheng, Cheng Tan

**Affiliations:** 1 Aerospace Information Research Institute, Chinese Academy of Sciences, Beijing 100190, China; heding201@mails.ucas.ac.cn (D.H.); dukaiyuan18@mails.ucas.ac.cn (K.D.); 2 School of Electronic, Electrical and Communication Engineering, University of Chinese Academy of Sciences, Beijing 101408, China; zzq_1633@163.com; 3 Naval Submarine Academy, Qingdao 266000, China; qdqyyzt@163.com (Z.Y.); grass2009@126.com (J.C.); hagooddemon@163.com (P.C.); 4 Institute of Information Engineering, Chinese Academy of Sciences, Beijing 100085, China

**Keywords:** digital step attenuator, GaAs, low insertion loss, modified simplified T-type, phase error compensation

## Abstract

In a beamforming circuit for a modern broadband phased-array system, high accuracy and compactness have received sufficient attention as they are directly related to side lobe level and fabrication cost, respectively. In order to meet the low phase error required, this paper proposed an ultra-broadband 6-bit digital step switched-type attenuator (STA) with capacitive/inductive compensation networks. Compared to the conventional methods, the proposed technique employs an improved simplified T-structure with capacitive compensation networks, which simultaneously achieves low insertion loss and high-accuracy amplitude/phase control. In addition, on-chip level shifting circuit is integrated to avoid complex control schemes. The strategy of prioritizing return loss is adopted to alleviate the performance degradation caused by impedance mismatch after cascade. As a proof-of-principle demonstration, a wideband 6-bit STA with core area of only 0.5 mm × 1.8 mm was designed via 0.15-micrometer GaAs pHEMT technology. It exhibits ultra-broadband operation with a 31.5 dB amplitude tuning range and a 0.5 dB tuning step. The insertion loss of the reference state is 4–5.3 dB. The return loss is better than 15 dB for all the 64 states. The RMS amplitude and phase errors are less than 0.2 dB and 2° over the 10 to 20 GHz.

## 1. Introduction

Phased arrays are widely adopted in modern radio-frequency (RF)-integrated systems such as radar remote sensing and low-orbit broadband satellite communication applications due to their high-precision beam pointing, fast beam synthesis and scanning capabilities [1,2]. These superior characteristics result from the transmit/receive (T/R) components in each antenna unit. As one of the critical modules in phased-array T/R design, the amplitude control circuits play an indispensable role in tuning the link gain variation and suppressing the sidelobes [3,4,5,6]. Typically, its realizations can be classified as active-type [7] and passive-type categories [8], which should be determined on the basis of system specifications and performance requirements, including insertion loss (IL), resolution, tuning range, operating bandwidth, amplitude/phase error, and compactness. Compared to the method using a variable gain amplifier (VGA), the main-stream solutions prefer digital-step attenuator (DSA) since it offers high linearity, high switching speed, bidirectional wideband operation and fine amplitude control without any power consumption [9,10]. Currently, there is still a wide demand for single DSA designs for the following applications: (1) novel topologies specifically studied for DSAs to improve their RF performance [6,8,11], which do not need to be integrated with other functional chips; (2) Off-chip integration is required for wideband multimode multichannel receiver systems where several narrowband signals are multiplexed through a common attenuator; (3) Attenuator-first software-defined radios (SDRs) for communication infrastructure require a single MMIC DSA solution [12]; and (4) Customized DSA chip designs based on customer requirements for different bands.

**Figure 1:**
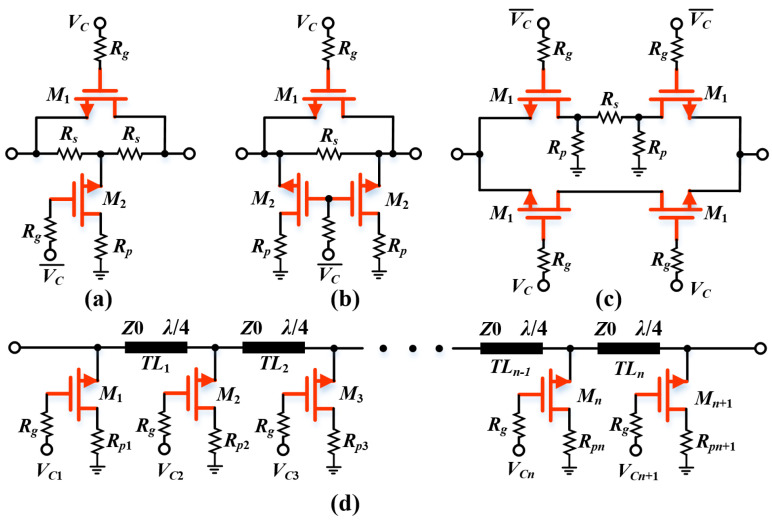
Typical schematics of attenuators (**a**) T-type; (**b**) π-type; (**c**) switched-path; (**d**) distributed.

Several common passive DSA topologies have been reported in the literature [11,12,13,14,15,16,17], including distributed [11,13], switched-path [14], and switched T-/π-/bridge T-type [15,16,17], as illustrated in Figure 1. These networks form a two-state architecture with selectable insertion paths through controlled RF switches, where the difference between the ILs of the respective states is the desired attenuation value [18]. Compared to the distributed structure exhibited in Figure 1d, other topologies with the advantages of a larger attenuation range and smaller chip area are preferred. However, with regards to a high-frequency broadband attenuator design, these structures critically suffer, gradually increasing amplitude/phase variations, which restrict the operating bandwidth of traditional DSAs [19]. Moreover, especially when cascading multi-stage units, the inter-stage impedance mismatch leads to further worsening of the property. In order to address this issue, the literature [20,21,22,23] elucidates the compensation methods and effects of π-type, T-type, bridge T-type and distributed structures, and verifies the principle under different technologies. Nevertheless, this solution still suffers from a high attenuator IL due to the presence of the transistor lossy resistance *R_on_* in the reference state, which is the reason why switching path designs should be adopted with caution [5]. To put it simply, this high-precision compensation approach comes at the cost of a high IL. Therefore, the realization of the amplitude tuning capability with accurate phase compensation while simultaneously maintaining compactness and low IL is an issue that needs to be addressed.

Furthermore, the characteristic of RF switches also constitutes majorly to the attenuator performance. These switches are achieved by utilizing CMOS [24,25], BJT/HBT [26,27], and HEMT [28,29] transistors. Among the numerous processes available, the GaAs pseudomorphic high-electron mobility transistor (p-HEMT) process is generally preferred due to its low switching loss and high isolation. Nevertheless, there is a serious concern that a negative voltage is required to control the on/off operation of the transistor, which is incompatible with conventional CMOS electrical level. Currently, to create positive voltage-controlled DSAs for better digital compatibility, novel realization schemes have been proposed in [30,31]. However, it affects the signals in the path and introduces resonance points at extremely low-frequency, which is not expected. Therefore, a more efficient form of positive voltage supply is highly demanded.

To address the above-mentioned issues, a novel positive voltage-controlled 6-bit DSA with low loss and high accuracy is proposed and designed in this paper, which mainly consists of several different structures, including improved simplified T-type, improved π-type, and switched-path. In contrast to conventional passive attenuators, the major contributions of the proposed DSA are as follows: (1) an improved and simplified T-type structure is utilized in place of the 0.5/1/2 dB attenuation units to provide low insertion loss and accurate phase compensation while maintaining a compact layout. (2) Aiming at the simplified T-type structure, the phase transfer characteristics as well as mathematical expressions for various compensation types at different frequencies are investigated and characterized for the first time, including the forms of series tail capacitor and shunt bypass capacitor. Moreover, the applicable attenuation range of the modified structure and the corresponding limiting factors are also analyzed and indicated. (3) the transfer function of the π-type unit is analyzed and the corresponding mathematical expressions are given to reveal the mechanism of bandwidth expansion. (4) In addition, the level shifting structure based on direct-coupled FET logic (DCFL) circuit is proposed to realize the positive voltage control of PHEMT transistor, which is favored to avoid a complex control signal loading scheme and exhibits a simple structure and fast speed with extremely low power consumption. Ultimately, this paper demonstrates a 10–20 GHz 6-bit DSA using 0.15-µm GaAs pHEMT technology.

## 2. The Adopted Technology

**Figure 2:**
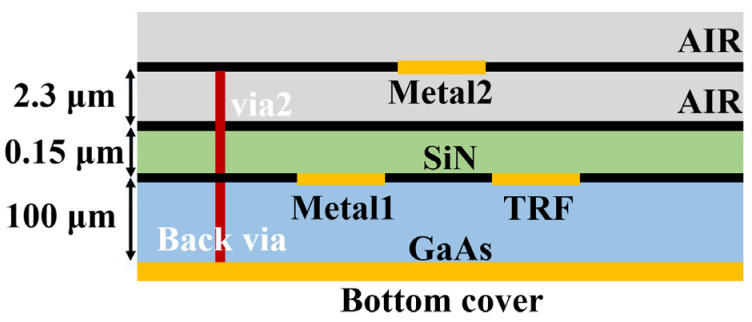
Cross-sectional view of the adopted GaAs technology.

The proposed design is based on the commercial 0.15 µm GaAs p-HEMT process from WIN Semiconductor Corp, whose cross-section on a GaAs substrate with a thickness of 100 µm and a dielectric constant of 12.9 is illustrated in Figure 2. It consists of air and SiN layers (with a dielectric constant of 6.9) with thicknesses of 0.15 µm and 2.3 µm, respectively. Two metal layers, from the top to the bottom, Metal-2, and Metal-1, have thicknesses of 4 μm and 1.33 μm, respectively. The technology provides thin-film resistors (TFRs) with a square resistance of 50 ohms and is available in a variety of transistor models, including coplanar waveguide (CPW) transistors, switching transistors, and E/D mode transistors (for level shifting circuits). The Metal–Insulator–Metal (MIM) capacitor can be realized by using via holes (via_2_) and double-metal layers. In addition, the high resistivity substrate, low loss tangent and high conductivity metal layer can effectively reduce the dielectric loss and conduction loss of all the above circuits. Consequently, the process is ideally suited for MMIC designs with low noise amplifiers, mixers, attenuators and phase shifters.

## 3. Modified and Simplified T-Type Structure Designed for Low Attenuation Units

### 3.1. Analysis of the Mechanism Contributing to the Phase Error

**Figure 3:**
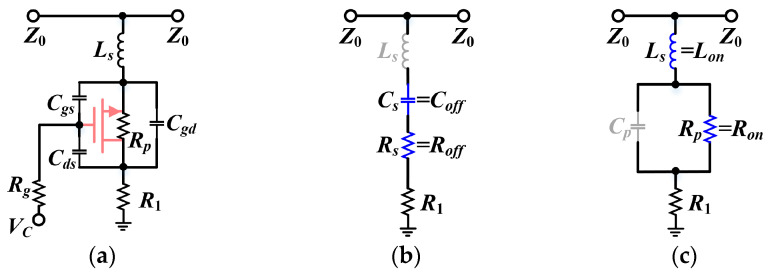
Typical schematics of the simplified T-type unit: (**a**) small-signal model; (**b**) equivalent circuit for reference mode with series *RC*s; and (**c**) equivalent circuit for attenuation mode with parallel *RC*s.

Among the various metrics of the DSAs, amplitude/phase characteristics, IL and voltage standing wave ratio have received the most attention. Although T-type and π-type structures can provide favorable control of attenuation accuracy with proper impedance matching, there is no avoidance of the extra path loss introduced by the transistor on-state resistance *R_on_*. In order to achieve a high-precision attenuation cell with low IL in a compact layout, we applied a simplified T-type cell, whose circuit model considering the parasitic parameters is illustrated in Figure 3a. To clarify the mechanisms jeopardizing the phase error of the unit branch, we start from the analysis of the proposed attenuator operated in reference mode and attenuation mode, respectively.

Figure 3b,c shows the equivalent circuits for the reference and attenuation states, respectively. As immediately seen, when turned off (i.e., reference mode), the transistor can be treated as a series connection of resistance *R_s_* (i.e., *R_off_*) and capacitance *C_s_* (i.e., *C_off_*), which are characterized by the transistor’s parasitic parameters as

(1) $$R_{s}=\frac{R_{p}{\left(C_{gd}+C_{gs}\right)}^{2}}{\omega^{2}{R_{P}}^{2}{\left(C_{gd}C_{gs}+C_{gd}C_{ds}+C_{gs}C_{ds}\right)}^{2}+{\left(C_{gd}+C_{gs}\right)}^{2}}$$

(2) $$C_{s}=\frac{C_{gd}C_{gs}+C_{gd}C_{ds}+C_{gs}C_{ds}}{C_{gd}+C_{gs}}+\frac{C_{gd}+C_{gs}}{\omega^{2}{R_{P}}^{2}\left(C_{gd}C_{gs}+C_{gd}C_{ds}+C_{gs}C_{ds}\right)}$$

where *ω* is the angular frequency of interest, and *R_p_*, *C_gs_*, *C_gd_* and *C_ds_* represent the parasitic resistance, gate-to-source parasitic capacitance, gate-to-drain parasitic capacitance and drain-to-source parasitic capacitance of the transistor, respectively. In this case, since the *C_off_* is extremely small, it is the dominant component contributing to isolation between the RF signal to ground. As a result, the phase characteristics exhibit a low-pass effect, which can be calculated as the following equation:

(3) $$\theta_{ref1}=-\tan^{-1}\left(\frac{\omega C_{off}Z_{0}}{2+\omega^{2}C_{off}^{2}R_{off,all}\left(2R_{off,all}+Z_{0}\right)}\right)$$

where *Z*_0_ denotes the characteristic impedance and *R_off,all_* = *R_off_* + *R*_1_. On the other hand, when the transistor is turned on (i.e., attenuation mode), it can be treated as a parallel connection of resistance *R_p_* (i.e., *R_on_*) and capacitance *C_p_* according to the equivalence principle, which is derived as

(4) $$C_{p}=\frac{C_{gd}C_{gs}+C_{gd}C_{ds}+C_{gs}C_{ds}}{C_{gd}+C_{gs}}$$

At this point, the impedance of *C_p_* is considerably larger to negligible compared to *R_p_*, thereby the parasitic inductance *L_s_* (i.e., *L_on_*, which contains metal lines connected to the source and drain of the transistor) and *R_p_* play a major factor, causing a high-pass effect. Its equivalent phase in the attenuation mode is calculated as

(5) $$\theta_{att1}=\tan^{-1}\left(\frac{\omega L_{on}Z_{0}\left(R_{on,all}+Z_{0}\right)}{2\omega^{2}L_{on}^{2}Z_{0}+Z_{0}R_{on,all}\left(R_{on,all}+\frac{1}{2}Z_{0}\right)}\right)$$

where *R_on,all_* = *R_on_* + *R*_1_. In addition, it is worth mentioning that the values of the transistor’s parasitic parameters in the above equations are variable in the on/off state, leading to quite different behavior. From the comparison of Equations (3) and (5), it can be found that there are opposite phase responses in the reference and attenuation modes, as previously mentioned. Moreover, the phase error will worsen with increasing frequency, severely limiting the application in high-frequency broadband circuits. In other words, an important conclusion is revealed that the effect of inductance *L_on_* needs to be minimized or eliminated to optimize the phase error.

### 3.2. Proposed Structure of the Attenuator with Bypass Compensation Technology

**Figure 4:**
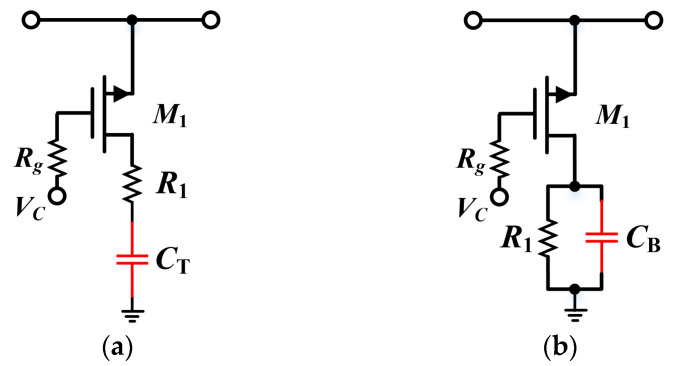
Schematic of the switched T-type attenuator cell: (**a**) modified structure with a series tail capacitor *C_T_*; (**b**) modified structure with a parallel bypass capacitor *C_B_*.

**Figure 5:**
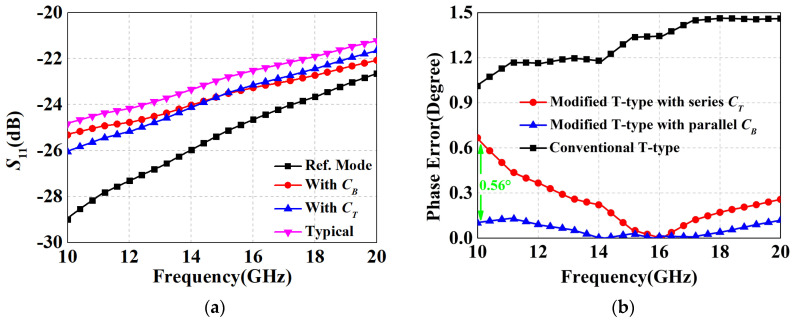
Simulated results: (**a**) *S*_11_; (**b**) phase error according to transistor on/off.

For phase correction architecture, the two simplest compensation solutions are adding additional series/shunt capacitors at the end of the branches. The modified switched T-type attenuator with a series tail capacitor *C_T_* is shown in Figure 4a. Depending on the equivalent circuit model, the transmission phases in the reference and attenuation modes, respectively, can be calculated as follows:

(6) $$\theta_{ref,seri}\approx -\tan^{-1}\left(\frac{C_{off}C_{T}\omega Z_{0}}{2\left(C_{off}+C_{T}\right)}\right)$$

(7) $$\begin{array}{cc} \theta_{att,seri}= & -\tan^{-1}\frac{\omega C_{T}\left(1-\omega^{2}C_{T}L_{on}\right)Z_{0}}{\omega^{2}C_{T}^{2}R_{on,all}\left(2R_{on,all}+Z_{0}\right)+2{\left(1-\omega^{2}C_{T}L_{on}\right)}^{2}}\\ & \approx -\tan^{-1}\frac{K\left(C_{T}\omega \right)}{L{\left(\omega C_{T}\right)}^{2}+M} \end{array}$$

where *K*, *L*, and *M* are ω-independent constants used to simplify the formula, since *ω*^2^ *C_T_ L_on_* is generally considered to be much less than 1. Based on Equations (6) and (7), it can be concluded that the presence of the tail capacitance significantly affects the transmission phase characteristics of the attenuation mode and changes its polarity. However, the phase effect on the reference mode is relatively insignificant since it has the same power in the numerator and denominator. Therefore, this method can only achieve phase compensation within a relatively narrow percentage bandwidth. Furthermore, using the same analytical approach, another compensation method using a parallel bypass capacitor *C_B_* is shown in Figure 4a, whose transmission phase function can be expressed as

(8) $$\theta_{ref,paral}\approx -\tan^{-1}\left(\frac{C_{off}\omega \left(C_{B}^{2}R_{p}^{2}\omega^{2}+1\right)}{2\left(C_{B}^{2}R_{p}^{2}\omega^{2}+2C_{off}C_{B}R_{p}^{2}\omega^{2}+1\right)}\right)$$

(9) $$\theta_{att,paral}\approx -\tan^{-1}\frac{O\left(C_{B}\omega \right)}{P{\left(\omega C_{B}\right)}^{2}+Q}$$

where *O*, *P*, and *Q* are also constants independent of *ω*. The above equations indicate that parallel capacitor *C_B_* can similarly modify the polarity of the attenuation mode and regulate its phase characteristics so as to make it as close as possible to the reference state behavior. Hence, this parallel form performs an improved broadband phase compensation characteristic compared to the tail capacitor, which shows consistency with the results in [32]. In order to demonstrate this difference more intuitively, simulation comparison experiments were performed and the corresponding results are illustrated in Figure 5.

Here, we take a 0.5 dB unit as an example. The switched transistor was selected as 1 × 15 µm since smaller sizes have less parasitic inductance and capacitances, which favoring higher bandwidth. From Figure 5b, it can be observed that the broadband compensation effect of *C_B_* is actually superior to that of *C_T_*, with the maximum variation decreasing from 0.67° to 0.09°. Hence, the parallel capacitor structure was finally used for the DSA design.

### 3.3. Design of 1 dB and 2 dB Attenuation Units with Low IL

**Figure 6:**
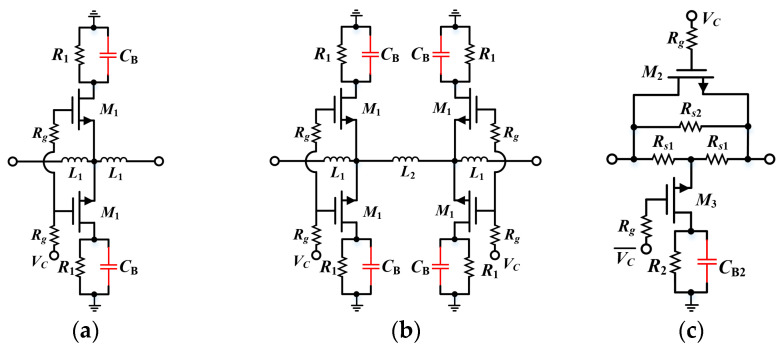
Schematics of (**a**) a 1 dB attenuator with modified simplified T-structure; (**b**) 2 dB attenuator with modified simplified T-structure; and (**c**) conventional bridge T-type 2 dB attenuator for comparison.

Since the proposed simplified T-type structure avoids the introduction of transistors in the on-state path, there is theoretically no resistor *R_on_* to cause any loss to the signal. Therefore, the modified structure was inherently allowed to maintain extremely low insertion loss performance across a wide bandwidth. In addition, the same technique was employed to form 1 dB and 2 dB attenuation cells in symmetrical arrangement. The corresponding schematics are presented in Figure 6a,b, respectively, where inductors *L*_1_, *L*_2_ were utilized to optimize the port matching.

**Figure 7:**
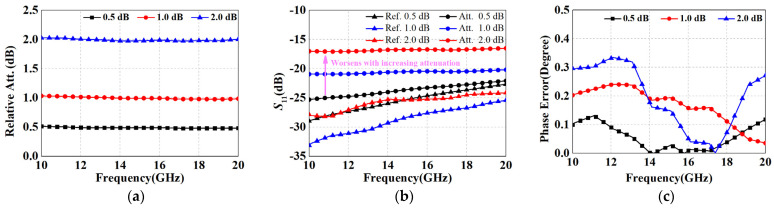
Simulation results for the proposed 0.5 dB, 1 dB and 2 dB attenuation structures: (**a**) relative attenuation; (**b**) *S*_11_; and (**c**) phase error.

Based on the RF performance simulation results in Figure 7, it can be shown that the 0.5 dB and 1 dB as well as the 2 dB unit all achieved accurate amplitude and phase control within 10–20 GHz, with maximum phase errors of 0.12°, 0.24°, and 0.34°, respectively. However, it is essential to note that this proposed approach of multiple parallel branches is not applicable to the large attenuation case. According to Figure 7b, the standing wave performance deteriorates significantly with increasing attenuation range, which will severely aggravate the amplitude/phase characteristics after cascading (generally, the *S*_11_ of each unit needs to be controlled below −15 dB). The above issue is due to the fact that parallel branches create a reduction in impedance that cannot be avoided. In addition, too many branches will consume more area and thus nullify the compactness advantage.

**Figure 8:**
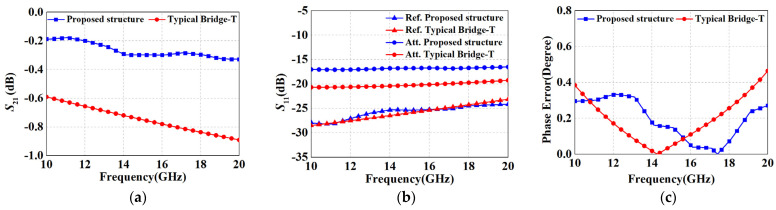
Comparative simulation results with traditional bridge T-type 2 dB attenuation structure: (**a**) *S*_21_; (**b**) *S*_11_; and (**c**) phase error.

Figure 8 demonstrates the performance comparison between the proposed structure and the conventional bridge T-type structure (as shown in Figure 7c) for 2 dB attenuation. It can be seen that the two architectures had quite close phase shift errors. Although the *S*_11_ of the proposed method was inferior to that of the conventional method, it was still maintained at −17 dB, which has less impact on performance after cascading. Additionally, based on the results in Figure 8a, the maximum IL of the proposed topology was only 0.32 dB, which is considerably lower than that of the conventional method (i.e., 0.9 dB), validating the effectiveness of the improved method. In conclusion, the proposed structure exhibited merits in the attenuation range below 2 dB, while other forms of topology were required for large attenuation cells.

## 4. High Attenuation Units and Level Shifter Structure Design

### 4.1. Analysis and Design of Modified π-Type and Switched-Path Type Structures for 4 dB, 8 dB and 16 dB Units

**Figure 9:**
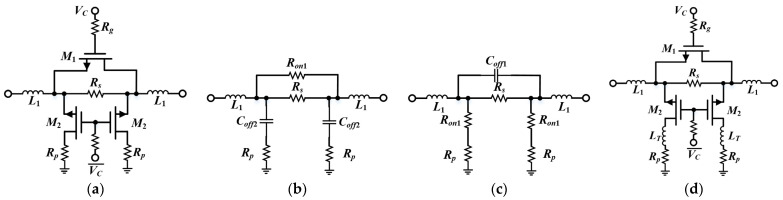
Schematics of (**a**) typical π-type structure1; (**b**) reference-state equivalent circuit; (**c**) attenuation-state equivalent circuit; and (**d**) modified π-type with series inductor.

**Figure 10:**
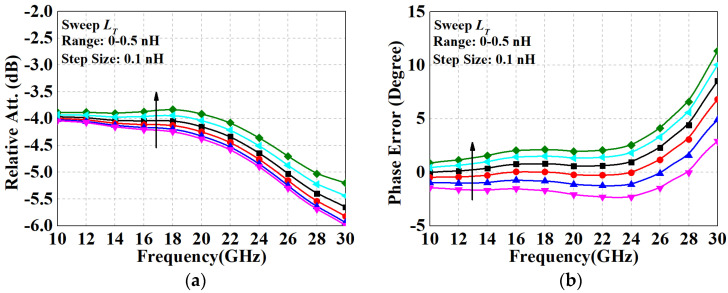
Analyzed (**a**) relative attenuation and (**b**) phase variation of the 4 dB π-type unit with various series inductances.

From the discussion in Section 3.3, it is clear that the proposed structure was no longer suitable for high attenuation cells. Instead, the π-type topology shown in Figure 9a was adopted as it has a larger bandwidth after compensation compared to the T-type topology. According to the reference and attenuation state equivalent circuits in Figure 9b,c, the transfer functions for their respective states can be derived as follows:

(10) $$T{\left(s\right)}_{\pi ,ref}=\frac{2Z_{0}{\left[1+C_{off2}R_{p}s\right]}^{2}}{\left(a_{1}s+1\right)\left(a_{2}s+b_{2}\right)}$$

(11) $$T{\left(s\right)}_{\pi ,att}=\frac{2Z_{0}\left(1+C_{off1}R_{s}s\right){\left(R_{p}+R_{on2}\right)}^{2}}{\left(R_{p}+R_{on2}+Z_{0}\right)\left(a_{3}s+b_{3}\right)}$$

where *a*_1_ = *C_off_*_2_(*R_p_* + *Z*_0_), *a*_2_ = *C_off_*_2_(*c*_2_*R_p_* + *Z*_0_*R_B_*), *b*_2_ = 2*Z*_0_ + *R_B_*, *R_B_* =*R_s_*||*R_on_*_1_, *a*_3_ = 2*C_off_*_1_*R_s_Z*_0_(*R_p_* + *R_on_*_2_), and *b*_3_ = *R_s_*(*R_p_* + *R_on_*_2_ + *Z*_0_) + 2 *Z*_0_(*R_p_* + *R_on_*_2_). It is well known that when zeros and poles occur at the same or a closely approximated frequency, they cancel out each other and thus produce a flat response. As can be learned from Equations (10) and (11), it is rather difficult to satisfy pole-zero cancelation relying solely on resistor selection and transistor sizing. However, when additional compensating components are introduced, more poles and zeros are tacked on to their numerator and denominator, resulting in more opportunities for tuning. Here, we applied the compensation scheme of the series inductor, as shown in Figure 9d. By analyzing the updated derived transfer function, it is able to find the critical zeros and poles to obtain a flat amplitude/phase characteristic. In order to visualize the effect and avoid complicated formulas, the simulated performance of the 4 dB unit with various *L_T_* is given in Figure 10. In view of the results, an appropriate increase in *L_T_* favored the bandwidth expansion of the relative attenuation, while an optimal compensation value existed for the phase error. Therefore, the value of *L_T_* needs to be considered as a tradeoff according to the actual situation. The initial values of the parameters adopted for the 4 dB and 8 dB units are shown in Table 1, which are relatively idealized components. Immediately, they should be further iteratively optimized based on the co-simulation performance after cascade design.

**Figure 11:**
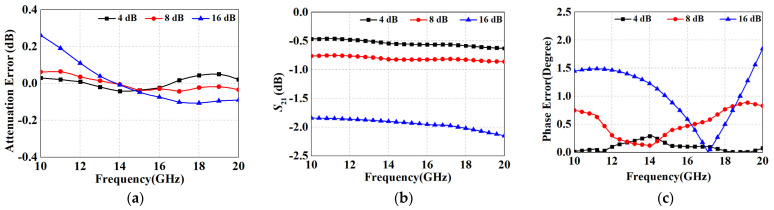
Simulation results for the proposed 4 dB, 8 dB and 16 dB attenuation structures (**a**) attenuation error; (**b**) *S*_21_; and (**c**) phase error.

As for the 16 dB unit, the attenuation is so large that either the π-type or T-type is no longer valid, so we employed the switched-path type in Figure 1c. The proposed performances of the 4 dB, 8 dB and 16 dB units are shown in Figure 11, exhibiting good accuracy in attenuation and phase error. However, their maximum ILs are 0.62 dB, 0.86 dB and 2.15 dB, which are significantly higher than those of the modified T-structure previously proposed in Section 3, and confirm the rationality of the modified scheme.

### 4.2. Level Shifter Structure

**Figure 12:**
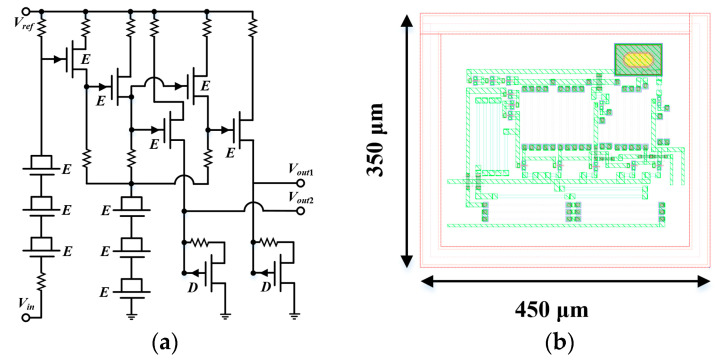
(**a**) Schematic and (**b**) layout of the level shifting circuit.

The inability to directly utilize positive voltages for on/off control of the transistor has shown poor digital compatibility for GaAs processes. Previous GaAs DSA designs have used off-chip serial peripheral interface (SPI) chips/devices to control the gate signals, which have the following drawbacks that have not yet been addressed: (1) additional gold bonding wires are required, which may lead to deterioration of RF performance and consistency; and (2) 6-bit DSAs demand 6-bit complementary controlled voltages, which means that there are a total of twelve off-chip power supply lines, twelve DC probes of 0/−5 V and corresponding on-chip pads for complexing the T/R modules, which dramatically increases the chip area and testing difficulty. In addition, the design of on-chip logic circuits should take into account both power consumption and cost. More complex GaAs-based serial-to-parallel (S/P) conversion modules, for example, have not been employed since their integration is much less than that of CMOS processes. In addition, p-HEMT transistors have, theoretically, gate parasitic diodes, which result in input logic thresholds that are sensitive to temperature (i.e., logic functions may fail at high temperatures), making the S/P modules difficult to be verified. In order to avoid the aforementioned disadvantages, this paper proposes an on-chip level-shifting circuit based on an integrated direct-coupled field-effect transistor logic (DCFL) structure with a compact layout of 450 µm × 350 µm, whose circuit schematic and layout are shown in Figure 12a,b, respectively.

**Figure 13:**
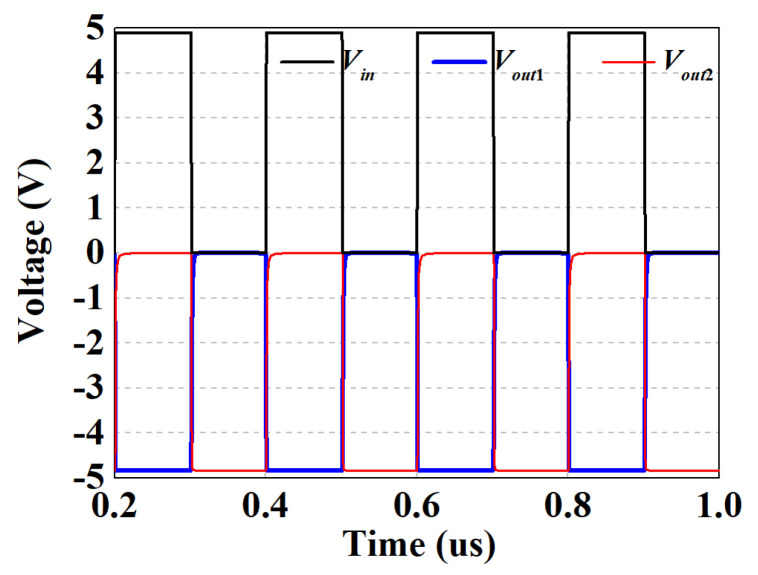
The time domain transient simulated results of the level shifting circuit.

The level shifter circuit is composed of voltage converters and inverters, which consists of E/D mode transistors (with opposite threshold voltage-temperature curves) to reduce temperature sensitivity and improve circuit stability. In addition, the diodes are formed by connecting the source and drain of the E-mode transistors. With the reference voltage *V_ref_* fixed at −5 V, the circuit converts the incoming 0/5 V pulses into a pair of complementary −5/0 V outputs simultaneously (i.e., *V_out_*_1_ and *V_out_*_2_), thus enabling positive voltage control of the ATT and saving half of the DC power lines and probes. The corresponding time domain transient simulation is illustrated in Figure 13.

## 5. Broadband 6-Bit High-Accuracy Digital Step Attenuator

**Figure 14:**
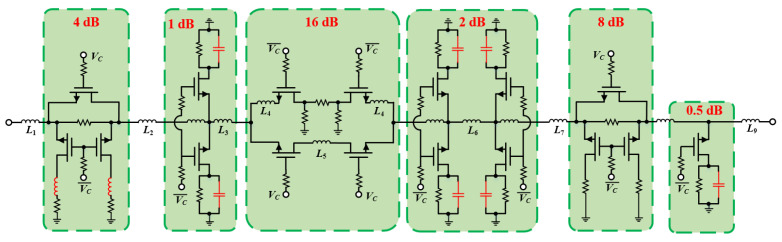
Schematic of the proposed ultrawideband high-accuracy DSA.

**Figure 15:**
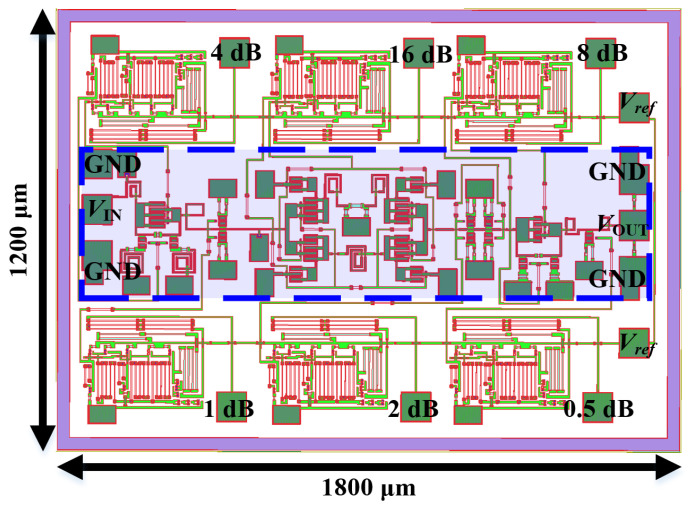
Overall view of the DSA with on-chip level-shifting circuit.

Combining the aforementioned broadband compensation technologies with an on-chip level shifter structure, a novel 6-bit 10–20 GHz high-accuracy DSA was proposed and designed using 0.15 µm GaAs pHEMT technology, and its schematic and corresponding layout are shown in Figure 14 and Figure 15, respectively. It has a total area of 1200 µm × 1800 um, including six on-chip integrated level shifting circuits and all RF/DC pads, with the ATT having a core area of only 520 µm × 1800 um. During cascading of the units, deterioration in matching conditions can contribute to variations in performance, and hence, all the initial parameters are optimized (according to the electromagnetic simulation results) over several iterations to ensure overall quality and stability. In addition, in order to make RF performance as close to the real situation as possible, the data for the on/off states of the switching transistors were measured and are provided by an on-wafer measurement system based on a four-port vector network analyzer Ceyear 3672E.

**Figure 16:**
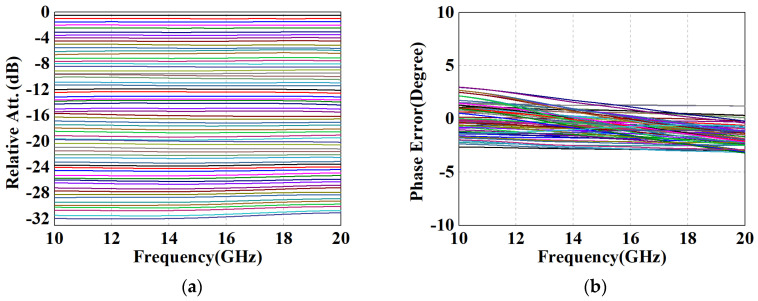
Performance results of the proposed DSA with measured switching transistors. (**a**) Relative attenuation and (**b**) phase error versus frequency for all the 64 attenuation states.

Figure 16a illustrates the relative attenuation for all 64 states, from which it can be seen that the attenuator has a dynamic range of 31.5 dB and an amplitude resolution of 0.5 dB. In addition, there is no overlap between these various states, which exhibits a favorable monotonic amplitude control performance. Figure 16b shows the phase variation of each state with respect to operation frequency. All curves were limited to within ±3° from 10 to 20 GHz, demonstrating precise phase control and verifying the validity of the proposed method.

**Figure 17:**
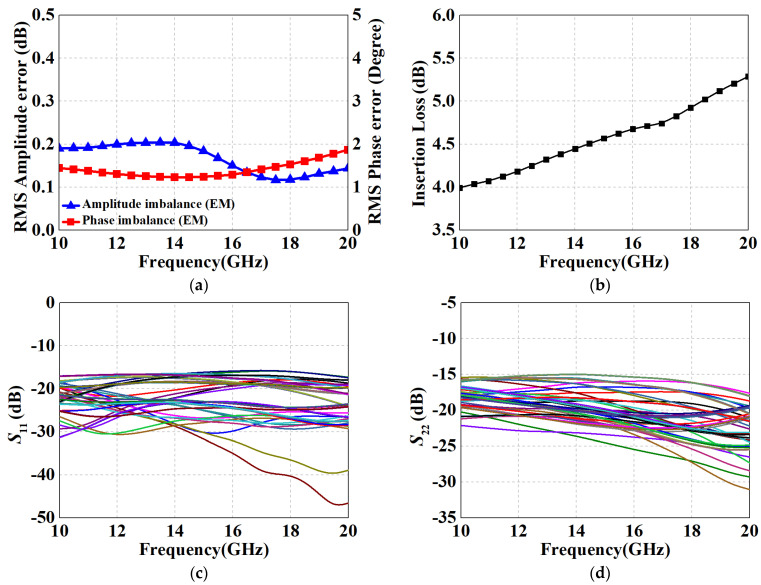
Performance results of the proposed DSA with measured switching transistors. (**a**) RMS amplitude errors; (**b**) IL at the reference state; (**c**) *S*_11_; and (**d**) *S*_22_.

As observed in Figure 17a, the root-mean-square (RMS) error of attenuation had a smooth response of 0.12 dB to 0.2 dB across the whole band, which also presented superior amplitude control. Moreover, it can be noticed that the maximum RMS phase error within the range of 10–20 GHz did not exceed 2°, which verifies the feasibility of the proposed structure for phase compensation. Figure 17b illustrates the IL of the design, i.e., *S*_21_ in the reference state. Profiting from the prominent performance of the simplified T-structure in the 0.5 dB to 2 dB units, the maximum IL is below 5.3 dB, with an average insertion loss of 4.65 dB. Moreover, according to the results of Figure 17c,d, the return losses of input and output better than 15 dB in all states (cascading causes a deterioration of standing waves, but still maintains the specification for engineering applications). Table 2 summarizes the performance of the designed broadband DSA and compared with other reported attenuators with state-of-the-art methods. Based on the reference data, it can be noticed that the proposed positive voltage control DSA has a more compact core circuit with the same or more attenuation units. Despite the addition of six on-chip logic circuits, the overall size remains within acceptable limits, achieving the desired goal of significantly reducing the phase error and insertion loss of the DSA without an appreciable increase in area.

## 6. Conclusions

In this paper, the phase characteristics of the simplified T-structure with different compensation networks were revealed and their applicable attenuation ranges and constraints were indicated. Due to the lossless behavior of the proposed structure on the path and the broadband phase compensation performance, the designed DSA achieved relatively low insertion loss and ultra-low phase error in the 10 to 20 GHz range. Additionally, on-chip level-shifting circuits were incorporated to avoid the traditional complex electrical control scheme and improve the reliability, making it ideal for highly integrated and broadband transceiver front-ends.

## Figures and Tables

**Table 1:** Parameter values for 4 dB and 8 dB units.

	*R_s_*	*R_p_*	*L*_1_	*L_T_*	*M*_1_	*M*_2_
4-dB	21.3 Ω	109 Ω	86.5 pH	0.315 nH	4 × 50 µm	1 × 15 µm
8-dB	35.3 Ω	13.5 Ω	67.5 pH	N/A	4 × 25 µm	1 × 15 µm

N/A Not applicable.

**Table 2:** Performance comparison with other attenuators.

Ref.	[2]	[33]	[34]	[35] ^#^	[28] ^#^	[36] ^#^	This work ^$^
Freq. (GHz)	DC-20	1–18	5–18	8.5–10.5	6–18	12–18	10–20
Integrated Control	No	No	No	No	Yes	Yes	Yes
Technology	0.13 µm BiCMOS	0.25 µm GaAs pHEMT	0.13 µm GaAs pHEMT	0.13 µm CMOS	0.25 µm GaAs pHEMT	0.18 µm GaAs pHEMT	0.15 µm GaAs pHEMT
Attenuation range (dB)	31.5 (6 bit)	15.5 (5 bit)	31.5 (6 bit)	30 (5 bit)	31.75 (7 bit)	15.5 (5 bit)	31.5 (6 bit)
IL (dB)	1.7–7.2	<5.7	<6.2	N.A.	<9	5–7.5	4.0–5.3
RL (dB)	>12	>10	>10	>11	>12	>12.5	>15
RMS amp. error (dB)	<0.37	<0.55	<1.1	<0.3	<0.6	<1	<0.2
RMS/Max phase error (°)	4/15	N.A./20	2/4	7/N.A.	5/7	2.2/N.A.	2/3
Area (mm^2^)	1.3 × 0.75	1.46 × 1.6	2.5 × 1.5	2.06 × 0.58	2.7 × 2	4.2 × 2.8	1.2 × 1.8 0.5 × 1.8 *

^#^ Multifunction Chip; * Core circuit area without pads and non-active space; **^$^** Simulation with measured transistors.

## Data Availability

The data that support the findings of this study are available from the author upon reasonable request.
